# Multi-omics Data Reveal SLC9A3R2 Promotes Breast Cancer Progression and an Immunosuppressive Microenvironment

**DOI:** 10.7150/jca.128052

**Published:** 2026-05-11

**Authors:** Zujin Chen, Yao Wang, Guixin Wang, Yue Yu, Xin Wang, Xuchen Cao

**Affiliations:** 1 The First Department of Breast Cancer, Tianjin Medical University Cancer Institute and Hospital, National Clinical Research Center for Cancer, Huan-Hu-Xi Road, He-Xi District, Tianjin, 300060 China.; 2 Key Laboratory of Cancer Prevention and Therapy, Tianjin, 300060 China.; 3 Tianjin's Clinical Research Center for Cancer, Tianjin, 300060 China.; 4 Key Laboratory of Breast Cancer Prevention and Therapy, Ministry of Education, Tianjin Medical University, Tianjin, 300060 China.

## Abstract

The molecular mechanisms driving the occurrence and progression of breast cancer remain unclear, necessitating the identification of novel molecular biomarkers and therapeutic targets. This study aims to investigate the role of *SLC9A3R2* (*NHERF2*) in breast cancer progression via an integrated multi-scale approach. Analysis of The Cancer Genome Atlas (TCGA) data revealed that *SLC9A3R2* is upregulated at both protein and mRNA level in breast cancer tissues, related to advanced tumor stage and poor prognosis, especially in luminal subtypes. Functional enrichment analyses linked high *SLC9A3R2* expression to epithelial-mesenchymal transition (EMT), estrogen response, and PI3K-AKT/MAPK signaling pathways. Additionally, *SLC9A3R2* expression was negatively correlated with the infiltration level of CD8+ T cell as well as the expression of four main immune checkpoint molecules. Single-cell RNA sequencing analysis of patient samples revealed that *SLC9A3R2*-positive tumor epithelial cells mediate aberrant cellular communication with endothelial cells, fibroblasts, and macrophages via specific ligand-receptor pairs involving *FN1, COL1A1*, and *COL1A2*. Cell functional assays showed that knockdown of *SLC9A3R2* significantly impaired the proliferation, migration, and invasion capacities of breast cancer cells. Our findings establish *SLC9A3R2* as a key promoter of breast cancer progression, influencing both intrinsic oncogenic pathways and the extrinsic tumor microenvironment (TME), which provides a novel insight for breast cancer treatment.

## Introduction

Breast cancer has remained a leading cause of cancer-associated mortality among women across the globe for several decades 1-3. Due to its considerable tumor heterogeneity, patient prognoses often vary significantly 4, 5. The development and application of molecular markers such as ER, PR, and HER2 have enabled more precise diagnosis and treatment stratification for breast cancer patients 6. However, even among patients with the same molecular subtype, responses to standard therapies can differ substantially. Moreover, the mechanisms driving breast cancer progression remain incompletely understood. Therefore, a persistent demand exists for uncovering new molecular biomarkers to overcome existing hurdles in the clinical management of breast cancer.

Breast cancer progression has been shown to involve multiple factors, including gene mutations, aberrant pathway activation, and remodeling of the tumor microenvironment (TME) 7, highlighting the importance of investigating drivers of breast cancer from a multi-omics perspective. Transcriptomic RNA sequencing enables the assessment of RNA expression at the tissue level, while single-cell RNA sequencing (scRNA-seq) provides resolution at the individual cell level 5. Integrating these two approaches can help elucidate the mechanisms underlying breast cancer progression from both tissue and cellular viewpoints.

*SLC9A3R2* (also known as *NHERF2*) is a nucleocytoplasmic scaffold protein. In the nucleus, it functions as a transcriptional co-regulator, while in the cytoplasm, it modulates the activity of G protein-coupled receptors (GPCRs) 8. Previous studies have reported that *SLC9A3R2* helps maintain endothelial homeostasis and may exert tumor-suppressive functions in certain cancer types 9, 10. However, a previous study reported *SLC9A3R2* contributes to tumor progression in estrogen-dependent breast cancer 11. Collectively, the existing literature presents conflicting findings, pointing to a dual, and potentially opposing, role for *SLC9A3R2* across various neoplastic contexts. Nevertheless, the functional impact and molecular mechanisms of *SLC9A3R2* in breast cancer remain poorly characterized and warrant further investigation.

In this study, we demonstrate a tumor-promoting role for *SLC9A3R2* in breast cancer. We found that *SLC9A3R2* is upregulated at both protein and mRNA level in breast cancer tissues, related to advanced tumor stage and poor prognosis, especially in luminal subtypes. Mechanistically, *SLC9A3R2* appears to be enriched in epithelial-mesenchymal transition (EMT) and other classical oncogenic pathways. In addition, *SLC9A3R2* expression was negatively correlated with the infiltration level of CD8+ T cell as well as the expression of four main immune checkpoint molecules. Furthermore, *SLC9A3R2* may regulate aberrant cellular communication involving *FN1, COL1A1*, and *COL1A2* with endothelial cells, fibroblasts, and macrophages. Finally, *in vitro* experiments confirm that knockdown of* SLC9A3R2* impairs the proliferation, invasion, and migration capacities of breast cancer cells. In summary, our study reveals a tumor-promoting function of *SLC9A3R2* and provides a theoretical basis for breast cancer.

## Methods

### Data Acquisition and Filtering

The TCGA-BRCA dataset was obtained from the official TCGA website. The inclusion criteria for samples were as follows: 1) availability of transcriptome sequencing data; 2) complete survival follow-up information and clinical staging data. Finally, 1,076 samples were collected for the further analyses. The single-cell dataset GSE180286, which contains scRNA-seq data from primary breast tumors and paired lymph nodes of five patients, was also utilized. Five primary breast cancer samples (1 luminal, 2 TNBC, 2 HER2+) were included for subsequent analysis.

### Differential Gene Expression and Clinical Characteristic Analysis

Based on tumor stage, TCGA-BRCA samples were categorized into stages I & II and stages III & IV. We employed the limma package 12 to identify differentially expressed genes, defining statistical significance by an adjusted P-value of less than 0.05. The UALCAN database was employed to investigate the clinical characteristics of the candidate gene *SLC9A3R2* in the TCGA-BRCA dataset, including sample type, tumor stage, and breast cancer subtype. Protein expression levels of SLC9A3R2 in normal breast tissue and breast cancer tissue were analyzed by The Human Protein Atlas (HPA). The prognostic significance of *SLC9A3R2* was assessed using Kaplan-Meier survival curves. A P-value < 0.05 was considered statistically significant.

### Functional Enrichment Analysis

The TCGA samples were divided by median value of *SLC9A3R2* mRNA level. Differential expression analysis was conducted using the limma package, with genes meeting adj. P < 0.05 considered differentially expressed. The Gene Set Enrichment Analysis (GSEA) and enrichment analyses (GO, KEGG) were performed using the clusterProfiler package 13. The P-values were corrected using the Benjamini-Hochberg (BH) method to control the False Discovery Rate (FDR).

### Immune Infiltration Analysis

TIMER 14 was utilized to analyze the correlation between SLC9A3R2 expression levels and immune cell infiltration (including neutrophils, B cells, CD4+ T cells, macrophages, dendritic cells, and CD8+ T cells) in TCGA-BRCA. Pearson correlation analysis was performed to assess the relationship between SLC9A3R2 expression and immune checkpoint molecules (*PDCD1, CD274, CTLA4, LAG3*). A P-value < 0.05 was considered statistically significant.

### Single-Cell Data Quality Control and Annotation

Five primary breast cancer scRNA-seq samples (GSM5457199, GSM5457202, GSM5457205, GSM5457208, GSM5457211) were subjected to stringent quality control thresholds to remove low-quality cells. The criteria included: 1) nFeature_RNA ≥ 200; 2) nCount_RNA ≥ 300; 3) percent_mito ≤ 20. Potential doublets were subsequently reduced using DoubletFinder 15. All samples were then merged, normalized, and integrated using Harmony 16 to correct for batch effects. The resolution of cell populations was determined based on principal component analysis (PCA) results. Cell clusters were annotated using well-established markers from published studies and publicly available databases 17-19.

### Cell-Cell Communication Analysis

Epithelial cells were categorized into *SLC9A3R2*-positive and *SLC9A3R2*-negative groups based on the detection of gene expression. Specifically, cells demonstrating a non-zero expression count for *SLC9A3R2* were defined as the *SLC9A3R2*-positive subpopulation. CellChat package 20 was applied to analyze intercellular communication across all cell types. The ligand-receptor pairs included in the analysis encompassed extracellular matrix (ECM), cell-cell contact, and secreted signaling pathways.

### Cell Culture, Transfection, and Cell Function Assays

The breast cancer cell lines CAL-51, T47D, MDA-MB-231, and MCF-7, along with the normal mammary epithelial cell line MCF-10A, were obtained from the American Type Culture Collection (ATCC, Manassas, USA). MCF-10A cells were maintained in MCF-10A-specific medium (Procell, China). CAL-51, T47D, and MDA-MB-231 cells were cultured following the conditions described in our previous study 17. All culture media were supplemented with 10% fetal bovine serum (FBS; Vazyme, Nanjing, China) and 1% penicillin/streptomycin (Gibco, USA). Cells were incubated at 37 °C in a humidified atmosphere containing 5% CO₂. Sequences of the siRNAs used in this study are provided in Supplementary File: Table S1. The experimental details for the functional cellular assays, including the Cell Counting Kit-8 (CCK-8) assay, colony formation assay, cell scratch assay, and Transwell invasion assay, are described in our previous publication 5, 17, 21. All experiments were repeat three times (*N* = 3).

### Reverse Transcription-Quantitative PCR Analysis

*SLC9A3R2* and *GAPDH* primers were synthesized by GentleGen (Suzhou, China), and all specific sequences are listed in Supplementary File: Table S2. The details of experiment were following our previous study.

### Statistical Analysis

The bioinformatics analyses were conducted by R (v4.3.0). Student's t-test was used for comparisons between two groups. Data were presented as mean ± standard deviation (SD), and *p* < 0.05 was considered significant.

## Results

### SLC9A3R2 is Associated with Breast Cancer Progression

**Figure 1:**
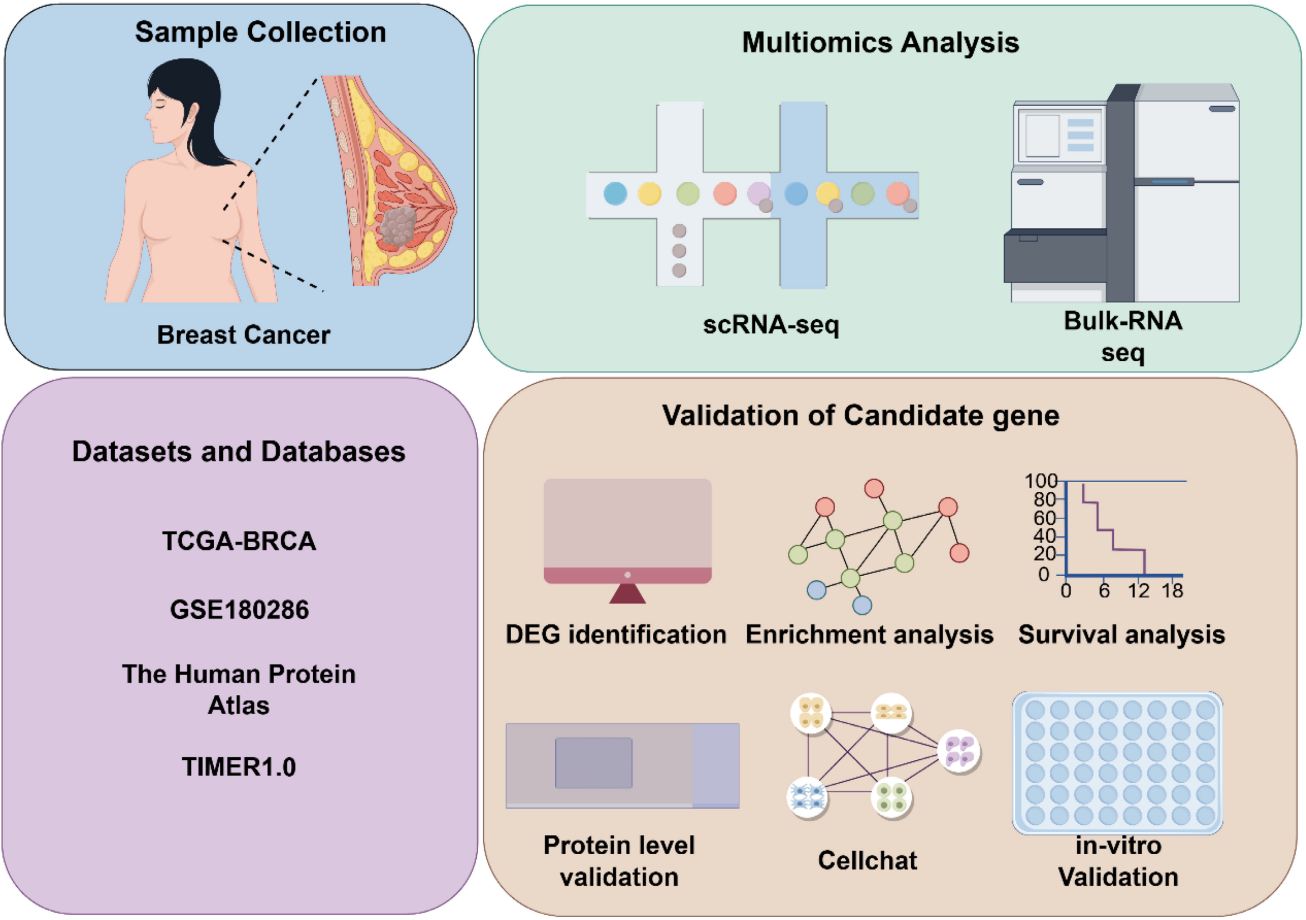
** Workflow of the study.** TCGA, The Cancer Genome Atlas; BRCA, breast cancer; GSE, Gene Expression Omnibus Series; TIMER, Tumor Immune Estimation Resource; DEG, Differential expressed genes; scRNA-seq, single-cell RNA sequencing.

**Figure 2:**
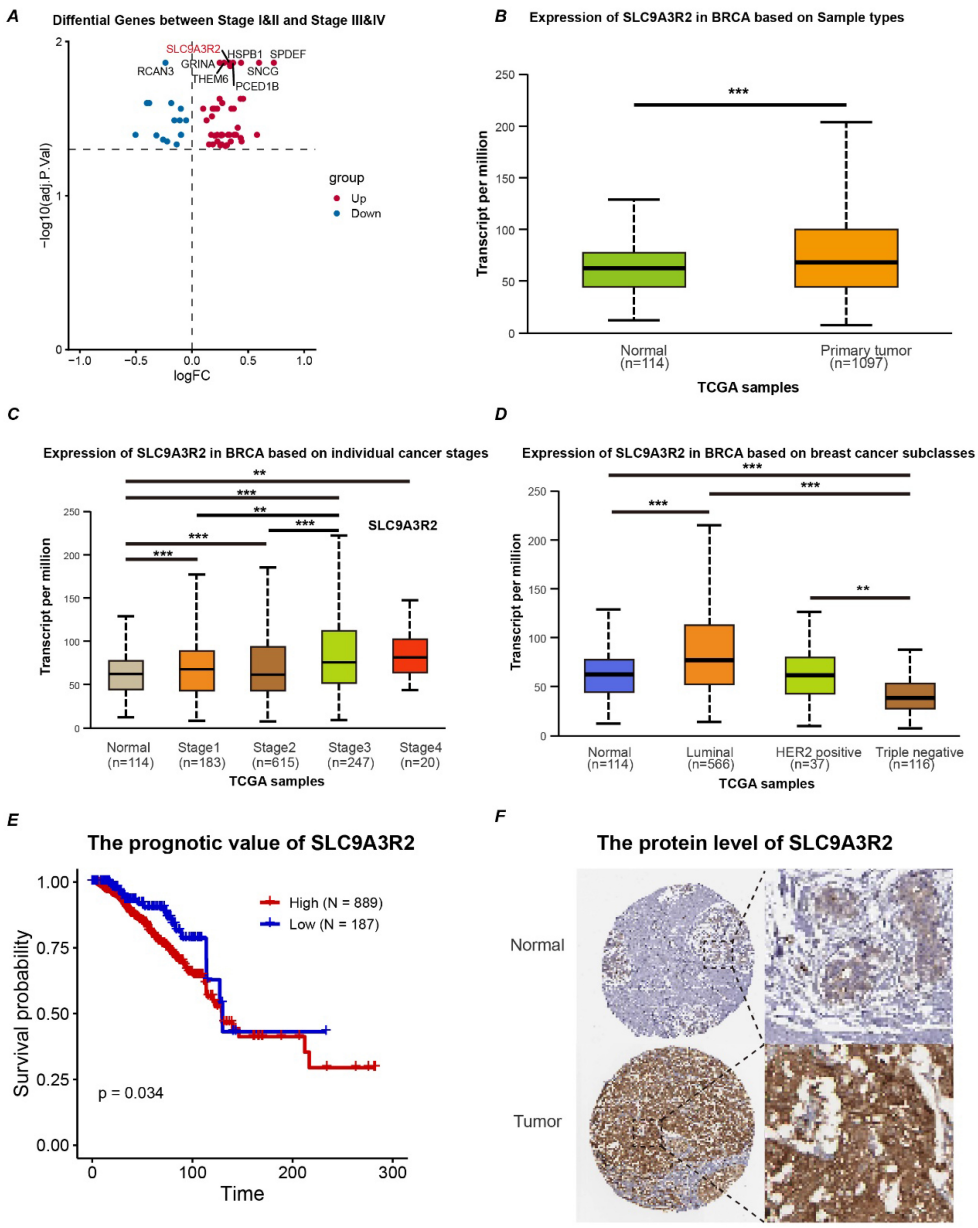
** SLC9A3R2 is upregulated in breast cancer tissues. (A)** The volcano map shows the differential genes between early and advanced breast cancer. **B-D** The box plot shows the expression level of SLC9A3R2 in tissues **(B)**, tumor stage **(C)** and molecular subtype **(D)**. **E** The Kaplan-Meier curves based on SLC9A3R2 mRNA level. **F** The protein level of SLC9A3R2 evaluated by Human Protein Atlas (HPA) database. **p < 0.01, ***p < 0.001

The overall study design is depicted in Figure 1. To identify key genes influencing breast cancer progression, we stratified breast cancer patients based on tumor stage. Using the limma package for differential expression analysis, 60 differentially expressed genes (DEGs) were identified. Specifically, 45 genes were upregulated and 15 were downregulated in the Stage III&IV group. Our analysis highlighted *SLC9A3R2* as among the genes demonstrating the most substantial and statistically significant upregulation (Figure 2A, Supplementary File: Table S3). Furthermore, the mRNA expression level of SLC9A3R2 was significantly higher in breast cancer tissues compared to normal breast tissues (Figure 2B), and its expression gradually increased with advancing tumor stage (Figure 2C). Interestingly, *SLC9A3R2* mRNA expression was highest in luminal breast cancer (Figure 2D), suggesting its potential as a diagnostic biomarker for hormone receptor-positive breast cancer. Assessment of patient survival revealed that elevated *SLC9A3R2* expression correlated with worse clinical outcomes (Figure 2E, p = 0.034). Similarly, at the protein level, *SLC9A3R2* expression was higher in breast tumor tissues than in normal breast tissues (Figure 2F). Collectively, these findings suggest that *SLC9A3R2* is upregulated at both the mRNA and protein levels in breast cancer tissues and could serve as a potential diagnostic biomarker. Additionally, its association with advanced tumor stage and poor prognosis indicates that *SLC9A3R2* is a potential prognostic marker for breast cancer.

### Downstream Signaling Pathways of SLC9A3R2

**Figure 3:**
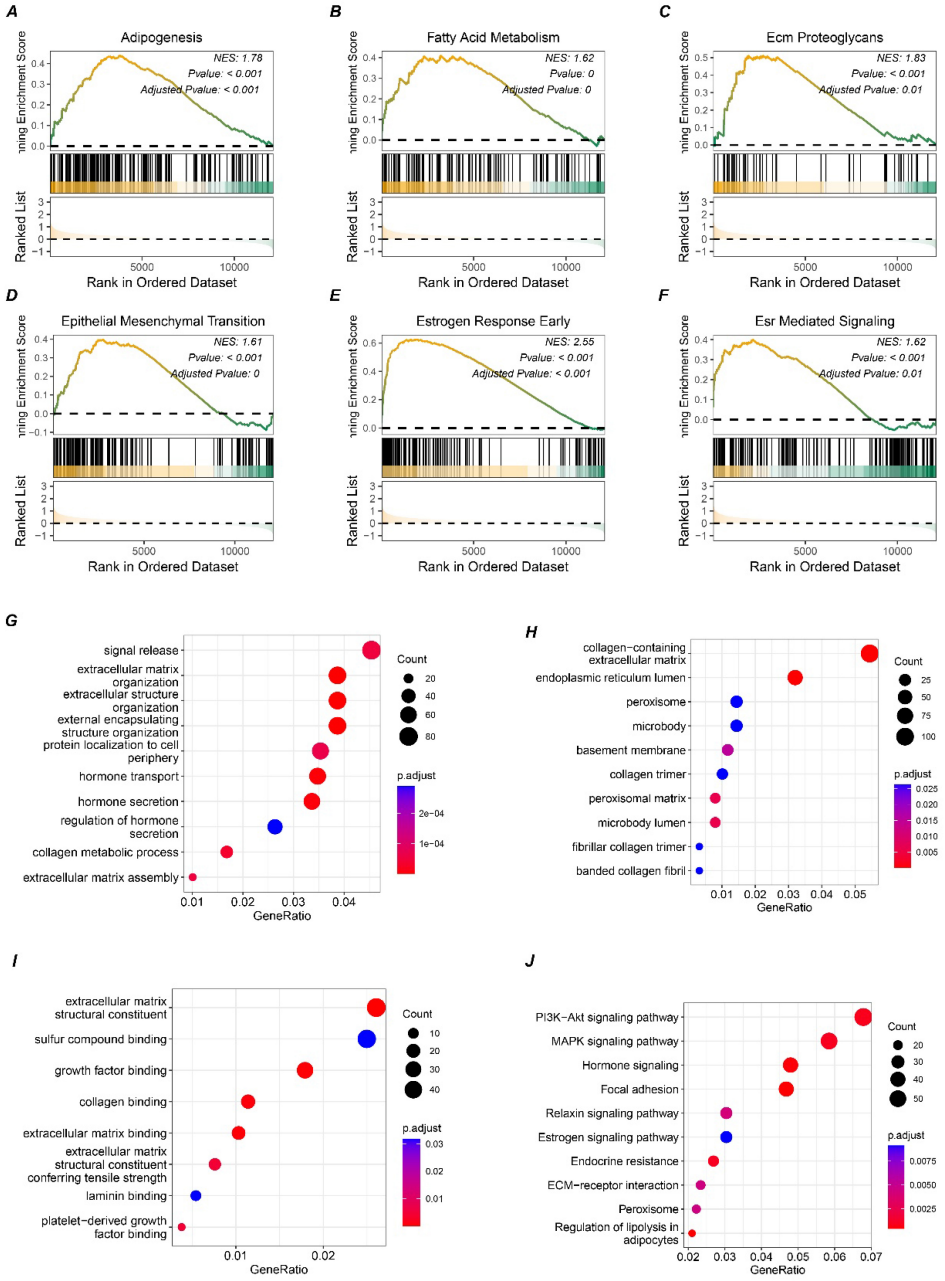
** Potential pathways associated with SLC9A3R2. A-F** GSEA results showing high expression of SLC9A3R2 was associated with adipogenesis **(A)**, fatty acid metabolism **(B)**, ECM proteoglycans **(C)**, epithelial-mesenchymal transition **(D)**, estrogen response early **(E)**, and ESR-mediated signaling **(F)**. **G-J** bubble diagrams showing the top 1500 highly expressed genes enriched in biological process **(G)**, molecular function **(H)**, cell component **(I)**, and KEGG **(J)**.

To further investigate the potential signaling pathways involving *SLC9A3R2*, differential expression analysis was performed to simulate the signaling pathways affected by *SLC9A3R2* expression perturbation. We noticed that elevated *SLC9A3R2* expression was related to pathways including adipogenesis (Figure 3A), fatty acid metabolism (Figure 3B), ECM proteoglycans (Figure 3C), epithelial-mesenchymal transition (Figure 3D), estrogen response early (Figure 3E), and ESR-mediated signaling (Figure 3F). These findings suggest that *SLC9A3R2* may promote tumor progression through metabolic reprogramming, extracellular matrix remodeling, and hormone signaling. Furthermore, we selected the top 1500 marker genes from the high *SLC9A3R2* group for Gene Ontology (GO) analysis. Consistent with the GSEA results, these genes were associated with the extracellular matrix and hormone signaling pathways (Figure 3G-I). KEGG enrichment analysis revealed that the top 1500 marker genes were involved in classic breast cancer progression pathways, including the MAPK, PI3K-AKT, and estrogen signaling pathway (Figure 3J), suggesting that *SLC9A3R2* may promote breast cancer progression through multiple oncogenic signaling pathways.

### Correlation Between SLC9A3R2 and Immune Infiltration

**Figure 4:**
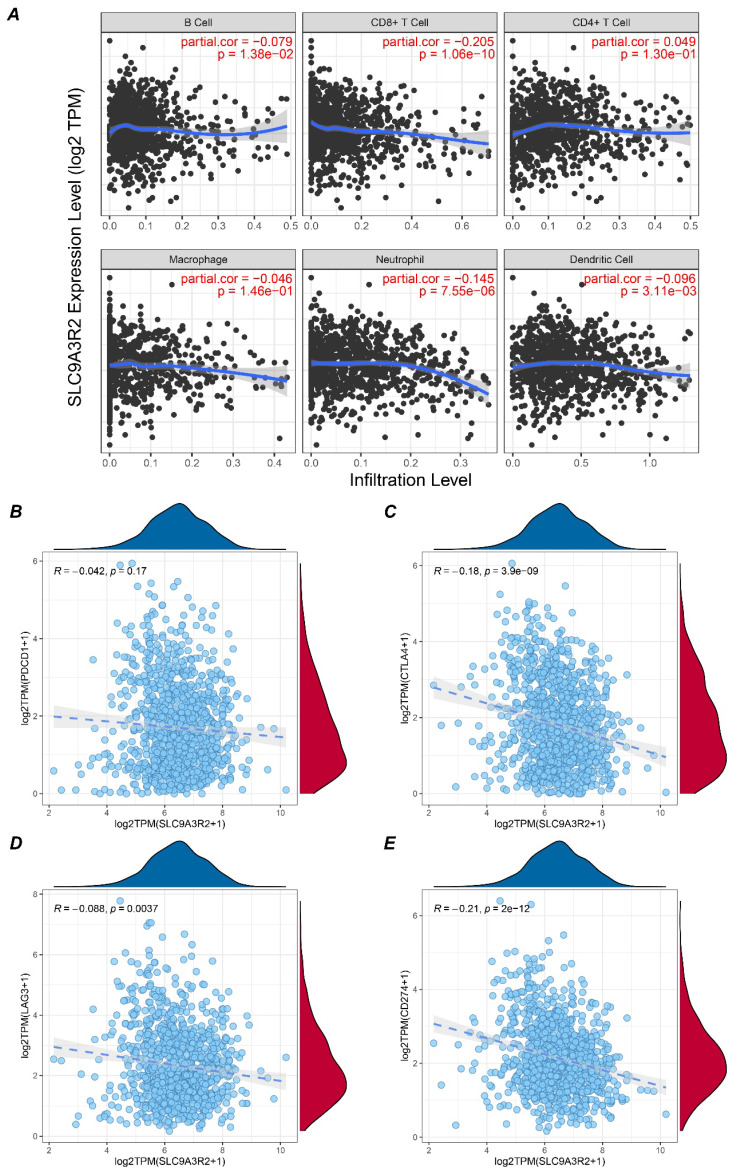
** Association between SLC9A3R2 and immune microenvironment. A** Correlation plots showing the expression level of SLC9A3R2 and B cell, CD8+ T cell, CD4+ T cell, macrophage, neutrophil, dendritic cell. **B-E** Scatter plots showing the correlations between SLC9A3R2 and PDCD1 **(B)**, CTLA-4 **(C)**, LAG3 **(D)**, CD274 **(E)**.

The aforementioned results preliminarily indicated a correlation between *SLC9A3R2* and breast cancer progression. We further analyzed its relationship with immune infiltration to elucidate the potential links between *SLC9A3R2* and the breast tumor microenvironment. Using the TIMER database, we analyzed the correlation between *SLC9A3R2* expression levels and the infiltration levels of six types of immune cells in breast cancer. As shown in Figure 4A, *SLC9A3R2* expression was significantly negatively correlated with CD8+ T cell infiltration (cor = -0.205, p = 1.06e-10). Given that CD8+ T cells are the primary effectors in tumor killing, this suggests that *SLC9A3R2* expression might contribute to poor prognosis in breast cancer patients by influencing CD8+ T cell infiltration. Furthermore, we observed no significant correlation between *SLC9A3R2* and *PDCD1* (Figure 4B, R = -0.042, p = 0.17), but significant negative correlations were found with *CTLA4* (Figure 4C, R = -0.18, p < 0.001), *LAG3* (Figure 4D, R = -0.088, p = 0.0037), and *CD274* (Figure 4E, R = -0.21, p < 0.001). These data imply that patients exhibiting high *SLC9A3R2* expression are unlikely to benefit from and may respond poorly to immune checkpoint blockade. In summary, our findings position *SLC9A3R2* as a regulator of the anti-tumor immune landscape, modulating both the expression of key immune regulatory molecules and the abundance of cytotoxic immune cells, highlighting its therapeutic implications for breast cancer immunotherapy.

### Immune Landscape of Breast Cancer

**Figure 5:**
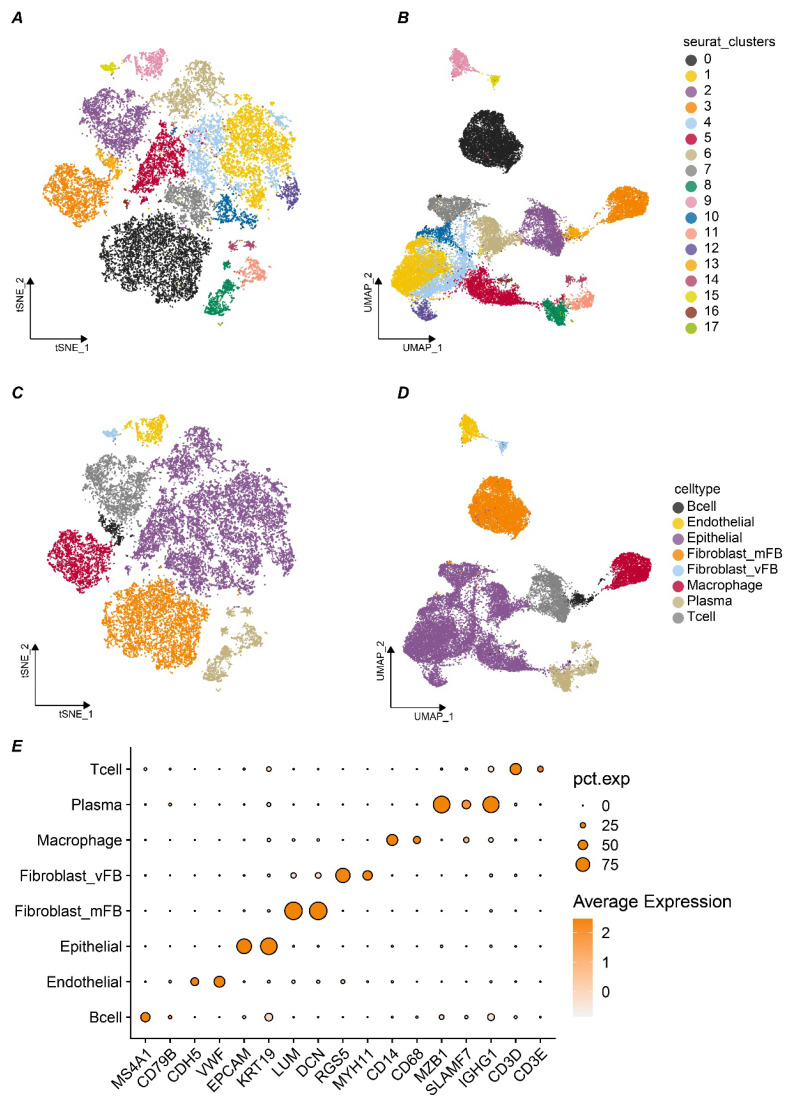
** The tumor microenvironment landscape of breast cancer at single cell level. A-B** t-distributed Stochastic Neighbor Embedding (tSNE) **(A)** and Uniform Manifold Approximation and Projection (UMAP) **(B)** scatter plots displayed 18 different cell clusters in breast cancer samples. **C-D** tSNE **(C)** and UMAP **(D)** scatter plots displayed 8 different cell clusters in breast cancer samples. **E** Dot plots showed expression levels of marker genes used to annotate 8 different cell types.

Given the pivotal role of cell-cell communication in tumor progression, elucidating the function of *SLC9A3R2* within this microenvironment becomes imperative. After doublet removal, normalization, batch effect correction, and dimensionality reduction of the five single-cell samples, a total of 26,440 high-quality single cells were obtained. At a resolution of 1.1, cell clustering stability was optimal, and 18 distinct cell clusters were identified (Figure 5A-B). Based on markers from published studies, these 18 clusters were annotated into eight different cell subtypes. As shown in Figure 5C-D, we successfully identified 426 B cells, 811 endothelial cells, 12,770 epithelial cells, 5,532 stromal fibroblasts, 206 vascular fibroblasts, 2,398 macrophages, 1,624 plasma cells, and 2,623 T cells. The markers we used were presented in Figure 5E. For instance, LUM and DCN were used for stromal fibroblasts, while EPCAM and KRT19 were used to annotate epithelial cells. In summary, we preliminarily identified eight major cell types within the breast cancer samples for subsequent analysis.

### SLC9A3R2 Mediates Aberrant Communication in the Breast Cancer Tumor Microenvironment

**Figure 6:**
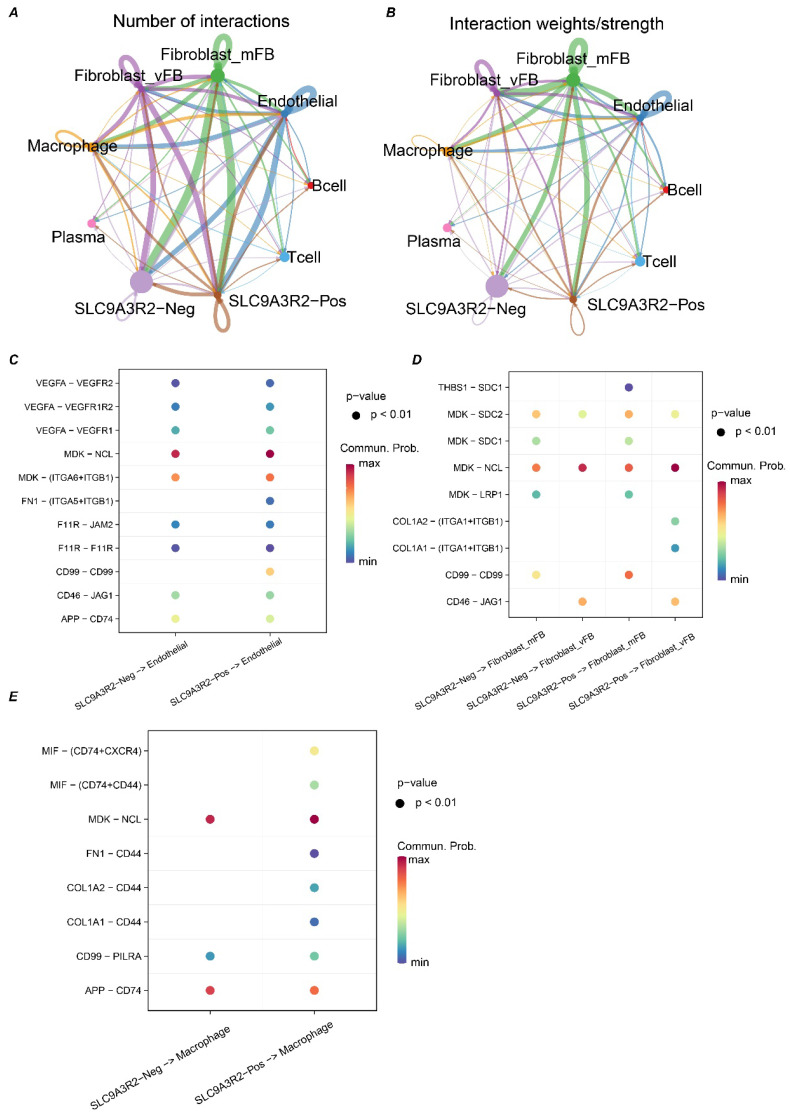
** The crosstalk between SLC9A3R2-positive/ SLC9A3R2-negative clusters and other components in tumor microenvironment. A-B** The number of interactions **(A)** and interaction weight **(B)** among all cell types in breast cancer microenvironment. The thickness of the lines represents the strength of the communication. **C** The interactions between SLC9A3R2-positive/ SLC9A3R2-negative cells and endothelial cells. **D** The interactions between SLC9A3R2-positive/ SLC9A3R2-negative cells and fibroblasts. **E** The interactions between SLC9A3R2-positive/ SLC9A3R2-negative cells and macrophages.

Previous results suggested an association between *SLC9A3R2* and the immune microenvironment of breast cancer. To gain deeper mechanistic insights, we next sought to delineate the precise contribution of *SLC9A3R2* to the breast tumor microenvironment. Since epithelial cells are the cellular origin of tumors, we stratified the 12,770 epithelial cells into *SLC9A3R2*-positive or -negative populations depending on whether they expressed this gene. We then performed cell-cell communication analysis across all cell types in TME to investigate the heterogeneity in communication between *SLC9A3R2*-positive/negative cells and other cells. We observed that the most active communications occurred between *SLC9A3R2*-positive/negative cells and stromal fibroblasts, vascular fibroblasts, endothelial cells, and macrophages (Figure 6A-B). Consequently, we further analyzed the communication heterogeneity among these cell populations. The *FN1-(ITGA-ITGB1)* and *CD99-CD99* ligand-receptor pairs showed specific interactions between *SLC9A3R2*-positive cells and endothelial cells, which were absent between *SLC9A3R2*-negative cells and endothelial cells (Figure 6C). The *THBS1-SDC1* ligand-receptor pair exhibited specific communication between SLC9A3R2-positive cells and stromal fibroblasts, compared to SLC9A3R2-negative cells and stromal fibroblasts (Figure 6D). The *COL1A2-(ITGA1+ITGB1)* and *COL1A1-(ITGA1+ITGB1)* ligand-receptor pairs showed specific interactions between *SLC9A3R2*-positive cells and vascular fibroblasts, unlike SLC9A3R2-negative cells and vascular fibroblasts (Figure 6D). These findings reveal potential signaling pathways mediated by SLC9A3R2 in tumor cells interacting with stromal cells. Additionally, the *(FN1, COL1A1, COL1A2)-CD44* ligand-receptor pair also showed specific interactions between *SLC9A3R2*-positive cells and macrophages (Figure 6E), suggesting that *SLC9A3R2* might mediate tumor cell-macrophage interactions via ECM-related signaling, thereby influencing the breast tumor microenvironment. In conclusion, these findings highlight the specific signaling interactions associated with *SLC9A3R2* in the breast cancer tumor microenvironment.

### Knockdown of SLC9A3R2 Inhibits Proliferation, Invasion, and Migration of Breast Cancer Cells

**Figure 7:**
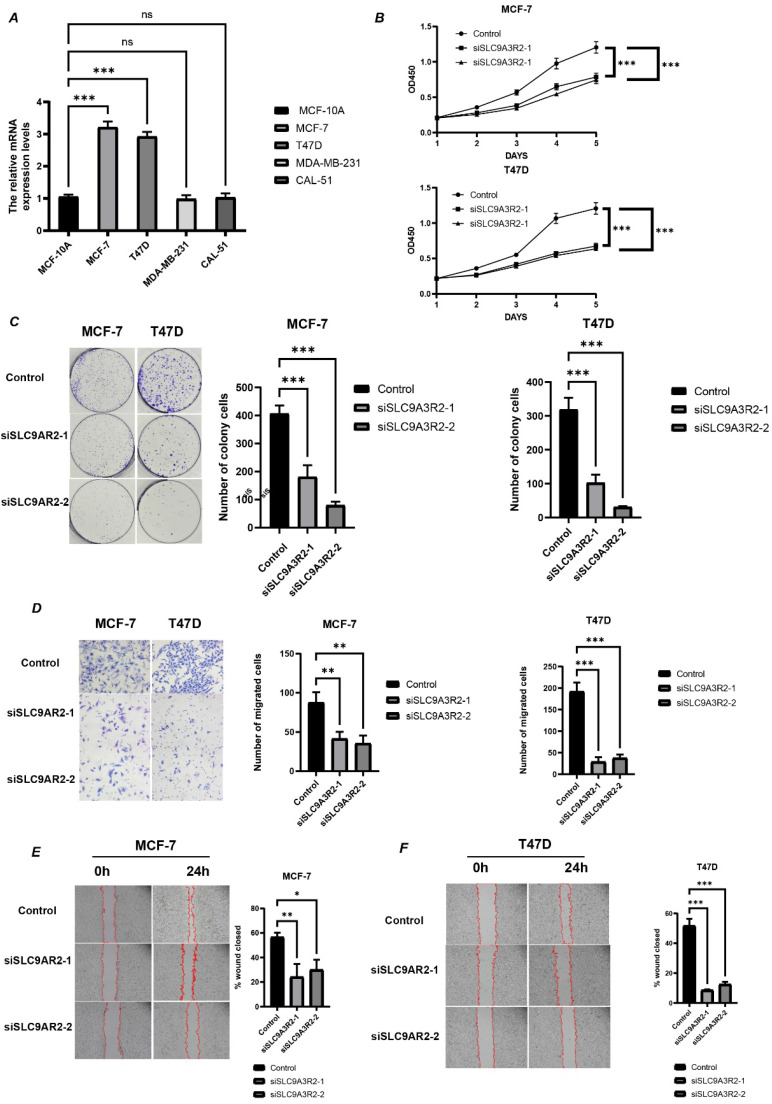
SLC9A3R2 was required for cell proliferation, migration, and invasion. **A** The mRNA level of SLC9A3R2 was detected by RT-qPCR in breast cancer cell lines. **B-C** The cell proliferation assays were conducted, including CCK-8** (B)** and colony formation assay** (C)**. **D-F** The invasion and migration assays were performed, including transwell assay **(D)** and scratch assay **(E, F)**. All experiments were repeat three times (*N* = 3). ns, no significance; *p < 0.05; **p < 0.01; ***p < 0.001.

Subsequently, we conducted *in vitro* experiments to validate our bioinformatics findings. First, we measured *SLC9A3R2* expression levels via RT-qPCR in a normal mammary epithelial cell line and four breast cancer cell lines (Figure 7A). *SLC9A3R2* mRNA expression was highest in MCF-7 and T47D cells, consistent with our previous findings. We then knocked down *SLC9A3R2* and performed cell proliferation-related assays. The CCK-8 assay results demonstrated that *SLC9A3R2* knockdown significantly inhibited the viability of MCF-7 and T47D cells (Figure 7B). Consistently, the colony formation assay confirmed that *SLC9A3R2* knockdown impaired tumor cell proliferative capacity (Figure 7C). Subsequently, the Transwell assay showed that the number of migrating MCF-7 and T47D cells was significantly reduced after *SLC9A3R2* knockdown (Figure 7D). Furthermore, the Wound Healing assay indicated that *SLC9A3R2* knockdown led to a significant reduction in the migration area of MCF-7 and T47D cells (Figure 7E-F). These findings demonstrate that inhibiting *SLC9A3R2* impairs the proliferative, invasive, and migratory capacities of breast cancer cell lines, identifying *SLC9A3R2* as a potential therapeutic target in breast cancer.

## Discussion

The mechanisms underlying breast cancer progression remain a major focus in breast cancer research 22, 23. However, given the high degree of tumor heterogeneity, studies based solely on tissue-level mRNA expression are insufficient to fully elucidate the contribution of the TME in disease development. Therefore, integrating scRNA-seq with bulk transcriptome analysis offers a more comprehensive strategy to uncover the underlying mechanisms. In this study, we combined multi-omics analyses with *in vitro* experiments to identify *SLC9A3R2* as a key molecule linking intrinsic tumor cell oncogenicity to remodeling of the immune microenvironment in breast cancer. To our knowledge, this is the first study to report the role of *SLC9A3R2* in the tumor immune microenvironment of breast cancer.

The reported functions of *SLC9A3R2* in cancer are indeed controversial. Lin et al. 24 found *SLC9A3R2* (*NHERF2*) interacts with *SLC26A3*, stabilizes IκB protein by counteracting its ubiquitination, and suppresses tumorigenesis and metastasis in colorectal cancer. In contrast, Yoshida et al. 25 reported that *SLC9A3R2* is upregulated in advanced colorectal cancer, where it promotes tumor progression partly via STAT3 and CD24. These findings suggest that *SLC9A3R2* may exert dual roles in cancer, potentially depending on the stage of tumor progression. Interestingly, *SLC9A3R2* has been identified as a co-activator of ERα, forming a complex with ERα and SRC-1 at the promoters of ERα target genes to enhance tumor cell proliferation and tumor growth in breast cancer 11. These studies indicated SLC9A3R2's function is fundamentally dictated by the specific oncogenic interactome of the host tissue and the spatiotemporal stage of tumor progression. Consistent with these reports, our results show that *SLC9A3R2* expression is related to higher tumor stage, poor clinical outcome, and highest expression in luminal breast cancer, indicating it could be served as a promising biomarker in hormone receptor-positive breast cancer. Furthermore, we also found that *SLC9A3R2* is linked to the PI3K-AKT and MAPK pathways, both well-established drivers of breast cancer proliferation, invasion, and metastasis. These observations imply that *SLC9A3R2* may promote breast cancer progression through activation of multiple oncogenic signaling pathways.

To further explore the role of *SLC9A3R2* in TME, we analyzed the relationship between *SLC9A3R2* and immune infiltration. Interestingly, *SLC9A3R2* expression was significantly negatively correlated with CD8+ T cell infiltration. Since CD8+ T cell is acknowledged as a main cell type to play an anti-tumor role in tumor, this finding provides an immunological basis for the poor prognosis associated with high *SLC9A3R2* expression. In addition, *SLC9A3R2* showed significant negative correlations with *CTLA-4* and *PD-L1* (*CD274*), indicating high-*SLC9A3R2* expression level patients might be less responsive to immune checkpoint therapies targeting *CTLA-4* or *PD-L1*.

Recent advancements in multi-omics and single-cell technologies have further elucidated the critical role of mitochondrial homeostasis and bioenergetics in shaping the breast cancer landscape. For instance, investigations into mitochondrial dynamin-like GTPase have highlighted its theragnostic value and its role in modulating the immune microenvironment, paralleling our findings on SLC9A3R2-mediated immune suppression 26. Furthermore, the remodeling of the TME is intricately linked to mitochondrial calcium gatekeepers, which regulate cellular signaling and contribute to breast cancer progression 27. Our observation that SLC9A3R2 correlates with metabolic pathways such as fatty acid metabolism is consistent with the discovery of the ATP synthasome's contribution to efficient energy flux in malignant breast cells 28. Together, these studies suggest that SLC9A3R2 may cooperate with mitochondrial regulatory networks to provide the energetic and metabolic foundation required for breast cancer invasion and the establishment of an immunosuppressive niche.

To gain deeper insight into how *SLC9A3R2* influences intercellular crosstalk in the tumor microenvironment, we performed cell-cell communication analysis. Interestingly, *SLC9A3R2*-positive tumor cells specifically interacted with endothelial cells via FN1 signaling, with fibroblasts via collagen and THBS1 pathways, and with macrophages through FN1 and collagen-mediated communication. This indicates that *SLC9A3R2* may act as an upstream regulator of *FN1* and collagen-related signaling. *COL1A1*-integrin signaling is reported to play an important role in mechanical signal transduction 29, suggesting that *SLC9A3R2* may promote invasion by altering the biomechanical tumor microenvironment. *THBS1-SDC1* signaling is involved in TGF-β pathway activation 30 and cancer-associated fibroblast (CAF) interactions, indicating a potential role for *SLC9A3R2* in stromal fibroblast activation. Moreover, CD44, a receptor on macrophages, signals through the PI3K/AKT pathway and has been linked to M2 polarization 31, 32, implying that *SLC9A3R2* may facilitate an immunosuppressive microenvironment by promoting M2-like macrophage polarization. Together, these findings uncover potential mechanisms by which *SLC9A3R2* shapes the tumor microenvironment, providing new insights and a theoretical foundation for future microenvironment-targeted therapies in breast cancer. Finally, we validated the pro-tumorigenic role of *SLC9A3R2* using *in vitro* models. Knockdown of *SLC9A3R2* significantly impaired proliferation, invasion, and migration in hormone receptor-positive breast cancer cells. These functional assays support our bioinformatic analyses and reinforce the potential of *SLC9A3R2* as a therapeutic target, particularly in hormone receptor-positive breast cancer.

Our study also has some limitations. The transcriptomic and single-cell datasets used here are relatively limited in sample size, which may introduce bias. Future studies involving larger cohorts are needed to validate our findings. Second, although our *in vitro* experiments offer foundational functional insights, these findings warrant further validation through *in vivo* animal models. Future investigations should also incorporate immune co-culture systems to functionally elucidate the predicted immune interactions, as well as co-immunoprecipitation assays to map the direct molecular binding events. Furthermore, given the pathway enrichment results, we plan to investigate the precise molecular mechanisms by *SLC9A3R2* modulates the PI3K/MAPK signaling cascades to promote tumor progression. These mechanistic studies will provide a deeper understanding of potential therapeutic targets.

## Supplementary Material

Supplementary tables.

## Data Availability

All the datasets in this study were obtained from public database including TCGA database (https://portal.gdc.cancer.gov/), GSE180286 (https://www.ncbi.nlm.nih.gov/geo/query/acc.cgi?acc=GSE180286).

## References

[1] Wagle NS, Nogueira L, Devasia TP, Mariotto AB, Yabroff KR, Islami F (2025). Cancer treatment and survivorship statistics, 2025. CA Cancer J Clin.

[2] Siegel RL, Kratzer TB, Giaquinto AN, Sung H, Jemal A (2025). Cancer statistics, 2025. CA Cancer J Clin.

[3] Siegel RL, Giaquinto AN, Jemal A (2024). Cancer statistics, 2024. CA Cancer J Clin.

[4] Polyak K (2007). Breast cancer: origins and evolution. J Clin Invest.

[5] Wang G, Shi C, He L, Li Y, Song W, Chen Z (2024). Identification of the tumor metastasis-related tumor subgroups overexpressed NENF in triple-negative breast cancer by single-cell transcriptomics. Cancer Cell Int.

[6] Loibl S, Poortmans P, Morrow M, Denkert C, Curigliano G (2021). Breast cancer. Lancet.

[7] Hong R, Xu B (2022). Breast cancer: an up-to-date review and future perspectives. Cancer Commun (Lond).

[8] Paredes-Villa AA, Aguilar-Arce IE, Meneses-Morales I, Cervantes-Roldan R, Valadez-Graham V, Leon-Del-Rio A (2025). NHERF2 Regulatory Function in Signal Transduction Pathways and Control of Gene Expression: Implications for Cellular Homeostasis and Breast Cancer. Arch Med Res.

[9] Lulic L, Jakovcevic A, Kovacic I, Manojlovic L, Dediol E, Skelin J (2023). HPV16 Impacts NHERF2 Expression in Oropharyngeal Cancers. Pathogens.

[10] Bhattacharya R, Wang E, Dutta SK, Vohra PK, E G, Prakash YS (2012). NHERF-2 maintains endothelial homeostasis. Blood.

[11] Meneses-Morales I, Tecalco-Cruz AC, Barrios-Garcia T, Gomez-Romero V, Trujillo-Gonzalez I, Reyes-Carmona S (2014). SIP1/NHERF2 enhances estrogen receptor alpha transactivation in breast cancer cells. Nucleic Acids Res.

[12] Ritchie ME, Phipson B, Wu D, Hu Y, Law CW, Shi W (2015). limma powers differential expression analyses for RNA-sequencing and microarray studies. Nucleic Acids Res.

[13] Yu G, Wang LG, Han Y, He QY (2012). clusterProfiler: an R package for comparing biological themes among gene clusters. OMICS.

[14] Li T, Fan J, Wang B, Traugh N, Chen Q, Liu JS (2017). TIMER: A Web Server for Comprehensive Analysis of Tumor-Infiltrating Immune Cells. Cancer Res.

[15] McGinnis CS, Murrow LM, Gartner ZJ (2019). DoubletFinder: Doublet Detection in Single-Cell RNA Sequencing Data Using Artificial Nearest Neighbors. Cell Syst.

[16] Korsunsky I, Millard N, Fan J, Slowikowski K, Zhang F, Wei K (2019). Fast, sensitive and accurate integration of single-cell data with Harmony. Nat Methods.

[17] Wang G, Cao J, Lu C, Cao Y, Wang S, Chen Z (2025). Multiomics profiling identifies the poor prognostic role of a tumor cluster with GNA15 overexpression in triple-negative breast cancer. Front Immunol.

[18] Wang G, Wang S, Song W, Lu C, Chen Z, He L (2025). Integrating multi-omics data reveals the antitumor role and clinical benefits of gamma-delta T cells in triple-negative breast cancer. BMC Cancer.

[19] Wang G, Chen Z, Tian Y, Zhu Y, Wang S, Song W (2025). Multi-Omics Profiling Identifies a High-Risk Subgroup of Breast Cancer Stem Cells for Prognostic Stratification and Personalized Treatment. J Cancer.

[20] Jin S, Guerrero-Juarez CF, Zhang L, Chang I, Ramos R, Kuan CH (2021). Inference and analysis of cell-cell communication using CellChat. Nat Commun.

[21] Chen ZH, Tian Y, Zhou GL, Yue HR, Zhou XJ, Ma HY (2023). CMTM7 inhibits breast cancer progression by regulating Wnt/beta-catenin signaling. Breast Cancer Res.

[22] Scimeca M, Urbano N, Bonfiglio R, Duggento A, Toschi N, Schillaci O (2019). Novel insights into breast cancer progression and metastasis: A multidisciplinary opportunity to transition from biology to clinical oncology. Biochim Biophys Acta Rev Cancer.

[23] Dominguez-Cejudo MA, Gil-Torralvo A, Cejuela M, Molina-Pinelo S, Salvador Bofill J (2023). Targeting the Tumor Microenvironment in Breast Cancer: Prognostic and Predictive Significance and Therapeutic Opportunities. Int J Mol Sci.

[24] Lin C, Lin P, Lin H, Yao H, Liu S, He R (2023). SLC26A3/NHERF2-IkappaB/NFkappaB/p65 feedback loop suppresses tumorigenesis and metastasis in colorectal cancer. Oncogenesis.

[25] Yoshida M, Zhao L, Grigoryan G, Shim H, He P, Yun CC (2016). Deletion of Na+/H+ exchanger regulatory factor 2 represses colon cancer progress by suppression of Stat3 and CD24. Am J Physiol Gastrointest Liver Physiol.

[26] Tzeng YT, Chu PY, Yong SB, Hsu TS, Tseng LM, Hou MF (2024). Spatial and Single-Cell Investigations Illuminate Theragnostic value and Immune Landscape of Mitochondrial Dynamin-Like GTPase in Breast Cancer. J Cancer.

[27] Tzeng YT, Chu PY, Yong SB, Hsu TS, Tseng LM, Hou MF (2024). Multi-omic profiling of breast tumor microenvironment uncovers a role of mitochondrial calcium gatekeepers. J Cancer.

[28] Tzeng YT, Kang YT, Hsiao TH, Chu PY, Yong SB, Lin SC (2026). ATP synthasome contributes to efficient energy flux in malignant breast cancer. Mol Cancer.

[29] Hynes RO (2009). The extracellular matrix: not just pretty fibrils. Science.

[30] Cui J, Jin S, Jin C, Jin Z (2020). Syndecan-1 regulates extracellular matrix expression in keloid fibroblasts via TGF-beta1/Smad and MAPK signaling pathways. Life Sci.

[31] Su H, Na N, Zhang X, Zhao Y (2017). The biological function and significance of CD74 in immune diseases. Inflamm Res.

[32] You G, Zheng Z, Huang Y, Liu G, Luo W, Huang J (2023). scRNA-seq and proteomics reveal the distinction of M2-like macrophages between primary and recurrent malignant glioma and its critical role in the recurrence. CNS Neurosci Ther.

