# Supplementary material for: Multi-omics Data Reveal SLC9A3R2 Promotes Breast Cancer Progression and an Immunosuppressive Microenvironment
Source: J Cancer. 2026 May 11;17(5):1018–31. doi: 10.7150/jca.128052 (PMC13189848; doi:10.7150/jca.128052)
Supplement: Supplementary file 1 — Supplementary tables. [file jcav17p1018s1.pdf]

Table S1 oligonucleotide sequences of siRNA

	FSequence (5' to 3')	RSequence (5' to 3')
SiRNA-1	GAGACAGAUGAACACUUCATT	UGAAGUGUUCAUCUGUCUCTT
SiRNA-2	CCUGGUUCCGACAAGGACATT	UGUCCUUGUCGGAACCAGGTT

Table S2 The primers of GNA15 and GAPDH

SLC9A3R2-F	GGCCAGTACATCCGCTCTG
SLC9A3R2-R	CAGGTCACTTCGGGACGAG
GAPDH-F	ACAACTTTGGTATCGTGGAAGG
GAPDH-R	GCCATCACGCCACAGTTTC

Table S3. The DEGs between early and advanced breast cancer

	logFC	AveExpr	P.Value	adj.P.Val
HSPB1	0.436848	9.721764	1.84E-06	0.013717
SLC9A3R2	0.332005	6.431719	2.71E-06	0.013717
SPDEF	0.729395	7.699726	2.90E-06	0.013717
RCAN3	-0.23702	4.06702	3.43E-06	0.013717
PCED1B	0.365568	3.964922	3.56E-06	0.013717
GRINA	0.246491	8.774259	4.55E-06	0.013717
SNCG	0.597217	4.797785	4.93E-06	0.013717
THEM6	0.286253	5.893176	5.50E-06	0.013717
INMT	0.339962	1.922754	6.51E-06	0.014423
SLC27A3	0.24592	4.160963	1.26E-05	0.023508
NQO1	0.432485	6.908993	1.39E-05	0.023508
GATA2	0.455585	3.159877	1.41E-05	0.023508
CD1A	-0.4021	1.218356	1.69E-05	0.025058
CLEC14A	0.269946	4.876009	1.85E-05	0.025058
ZNF121	-0.18531	4.182777	1.97E-05	0.025058
TTC22	-0.38124	2.498653	2.01E-05	0.025058
KRT18	0.350655	9.215539	2.51E-05	0.02726
NHLH1	-0.09975	0.445942	2.60E-05	0.02726
ABCB5	0.099516	0.211069	2.72E-05	0.02726
LYZL2	0.200675	0.390133	2.77E-05	0.02726
CLDN5	0.37239	2.995056	2.91E-05	0.02726
SERF2	0.193212	6.299102	3.12E-05	0.02726
LY6H	0.224511	1.06113	3.15E-05	0.02726
ZFYVE19	0.184303	4.618937	3.28E-05	0.02726
PXN	0.177754	4.24839	3.83E-05	0.030561
FASTKD1	-0.15722	3.533273	4.39E-05	0.032434
CCL14	0.129362	0.349952	4.69E-05	0.032434
SOX30	-0.10951	0.354705	4.70E-05	0.032434
CXorf58	-0.05267	0.236551	4.72E-05	0.032434
PODN	0.407378	4.63586	5.45E-05	0.036213

LTBP2	0.333044	5.85145	6.35E-05	0.040292
MPL	-0.09954	0.744767	6.54E-05	0.040292
SVEP1	0.320708	2.496199	7.19E-05	0.040292
ADRA2C	0.392419	2.237703	7.38E-05	0.040292
MAB21L1	0.224662	1.143123	7.49E-05	0.040292
GDPD3	0.375103	2.663911	7.66E-05	0.040292
LAMC2	-0.50346	4.372167	7.75E-05	0.040292
CYGB	0.258271	3.651759	8.31E-05	0.040292
KRT8	0.324619	9.035367	8.39E-05	0.040292
SPDYC	0.57898	1.362832	8.50E-05	0.040292
SOX18	0.267823	3.909763	8.54E-05	0.040292
CCM2L	0.170414	1.766235	8.58E-05	0.040292
SLFNL1	-0.09692	0.451478	8.69E-05	0.040292
BLVRB	0.270715	7.197406	8.92E-05	0.04041
CHPT1	-0.31724	5.09258	9.24E-05	0.04063
JAM2	0.259413	3.127438	9.37E-05	0.04063
VPS28	0.203343	6.840644	9.62E-05	0.04082
AMIGO2	0.433946	4.740779	0.000102	0.042257
GALNT13	-0.25791	0.830993	0.000106	0.043276
MMRN1	0.345395	1.707416	0.000113	0.044464
CYP27C1	-0.22151	0.586444	0.000116	0.044464
LRRC26	0.441718	2.180163	0.000118	0.044464
TIE1	0.22636	3.71624	0.000118	0.044464
TBXA2R	0.151772	2.084264	0.000128	0.046491
EXO5	-0.13673	2.499446	0.000129	0.046491
ABCB9	0.185852	1.972757	0.000132	0.046491
CYTL1	0.263728	1.98169	0.000133	0.046491
CXXC5	0.311154	6.454928	0.000137	0.046984
MMRN2	0.25201	4.42306	0.00014	0.047165
NR2F2	0.29922	5.545411	0.000143	0.047649
